# Epidemiological profile of kidney transplant patients with lupus nephritis

**DOI:** 10.1590/2175-8239-JBN-2024-0061en

**Published:** 2024-12-13

**Authors:** Beatriz Curto Pachi, Laura Marcon Bischoff Bialecki, Luísa Rigon Borba, Helena Marcon Bischoff, Valter Duro Garcia, Gisele Meinerz, Elizete Keitel

**Affiliations:** 1 Santa Casa de Porto Alegre, Porto Alegre, Rio Grande do Sul, Brazil.; 2 Universidade Federal de Ciências da Saúde de Porto Alegre, Porto Alegre, Rio Grande do Sul, Brazil.

**Keywords:** Systemic Lupus Erythematosus, Lupus Nephritis, Kidney Transplantation

## Abstract

**Introduction:**

Lupus nephritis (LN) affects up to 50% of patients with systemic lupus erythematosus (SLE) and may lead to kidney failure and require kidney transplantation (KT). Results compared to KT from other causes are controversial, and we aimed to assess the clinical course, complications, and survival of LN patients undergoing KT.

**Methodology:**

Retrospective cohort of 99 KT due to LN from 1977 to 2023 at a single center, divided into two groups according to the immunosuppression period: G1 (before 2009) and G2 (from 2009 onwards). Clinical and demographic characteristics, as well as clinical evolution, were compared.

**Results:**

Patients were predominantly white (65.9%), female (86.9%), in their first KT (83.8%). The median age was 20.0 (11.5–25.0) years at SLE diagnosis, and 30.0 (23.0–40.0) years at KT. Renal graft biopsy was indicated in 46% of patients, with rejection observed in 23%, and LN recurrence in 5%. When assessing the two distinct periods of standard immunosuppression, there was no difference in median glomerular filtration rate and proteinuria at 1 and 5 years, nor in 5-year survival. Throughout follow-up, 37.4% of patients lost their graft, and 13% died with a functioning graft. No graft loss was attributed to LN recurrence.

**Conclusion:**

KT is a successful treatment for LN, with graft survival rates similar to those of transplants from other causes. LN recurrence was not associated with renal graft loss.

## Introduction

Systemic lupus erythematosus (SLE) is a chronic systemic inflammatory disease of autoimmune origin, characterized by heterogeneous clinical presentations, and a course of remission and recurrence. There is great variability in the incidence/prevalence of this disease worldwide^1^. A Brazilian study estimated the incidence at 8.7 cases per 100,000 inhabitants^2^.

Lupus nephritis (LN) is the most common cause of kidney damage in SLE, occurring in 20% to 60% of patients^3,4^. Some factors may be associated with poorer renal prognosis, including Black and Hispanic ethnicities (compared to White), male sex, and HLA-DR3 and DR15 serotypes. In contrast, HLA-DR4 and DR11 serotypes seem to have a renal-protective role^3,5^.

Individuals with LN have higher mortality rates compared to patients without renal involvement, with results ranging from 5% to 25% within 5 years from disease onset^6^. In 10 years, over 50% of patients who partially respond to treatment and more than 85% of non-respondents may require renal replacement therapy^7^. These patients are candidates for kidney transplantation (KT), potentially exhibiting good graft function, low recurrence rates (2% to 11%)^3^, and better survival^8^, with fewer cardiovascular events and infections compared to LN patients who remain on dialysis^3^. Of particular interest, patients with a history of thrombosis or miscarriages should be evaluated for the possibility of antiphospholipid syndrome prior to transplantation to prevent perioperative complications^3^.

The present study aims to outline the epidemiological profile of LN patients who underwent transplantation at Santa Casa de Porto Alegre, evaluating their characteristics, clinical evolution, and graft survival after kidney transplantation. The literature review is controversial regarding the results when compared to transplants from other causes. This study aimed to assess the long-term outcome-associated factors in our population.

## Methods

### Design

Observational study of retrospective cohort.

### Sample

Patients who underwent kidney transplantation due to LN between January 1977 and June 2023, with follow-up until December 2023. The exclusion criterion was simultaneous transplantation of any other organ. If the same patient underwent more than one kidney transplantation in the period, each procedure was counted as a single case.

### Ethics Committee

The project was approved by the Institution’s Human Research Ethics Committee (protocol number 70505123.5.3001.5345).

### Data Collection

KT recipients were monitored by the team, and their demographic information, clinical and immunological characteristics, donor features, and follow-up status were updated in a database by the Nephrology Service, which is used to identify LN patients. Patients were considered to have LN when they were referred for KT with this diagnosis, without evidence of any other cause for kidney failure.

### Variables

Clinical, demographic, and laboratory characteristics of the recipients were investigated, along with donor data, immunosuppressive therapy, perioperative, cardiovascular and infectious complications, malignancies, and graft and patient survival. The glomerular filtration rate was calculated using the CKD-EPI 2021 equation based on serum creatinine, expressed in mL/min/1.73m^2^. Proteinuria was measured as the protein to creatinine ratio (PCR) in a urine sample. Extrarenal manifestations of SLE were identified according to medical records.

### Follow-up

Patients were followed up on an outpatient basis after KT, with monthly visits during the first year, followed by bi-monthly visits as long as renal graft function persisted. Renal function tests and urinalysis were collected at every visit. Renal biopsies were performed when clinically indicated, e.g. worsening renal function and/or proteinuria, rather than per protocol. Renal graft biopsies were analyzed at the hospital’s pathology laboratory.

### Immunosuppression

Immunosuppression protocols have changed over the study assessment period. The first years were based on azathioprine and prednisone, with the introduction of cyclosporine in the 1990s. Induction therapy became more widely used from the 2000s onwards, primarily based on basiliximab. From 2009 onwards, the primary protocol was based on tacrolimus, mycophenolate, and prednisone, with induction therapy on basiliximab or thymoglobulin for patients at higher immunological risk. Thus, we considered two periods of immunosuppression: G1, before 2009, based on cyclosporine, azathioprine and prednisone; and G2, from 2009 onwards, based on tacrolimus, mycophenolate, and prednisone, with increased use of induction therapy.

### Statistical Analysis

Categorical variables were described as absolute frequencies (n) and percentages (%), and compared using chi-square and Fisher’s exact test. Continuous variables were presented as measures of central tendency and dispersion, and compared with parametric and non-parametric tests, according to normal distribution. A significance level of 5% (p < 0.05) was considered for all statistical tests. Patient and renal graft survival were assessed by Kaplan-Meier, with significance determined by the log-rank test. Cox regression was used to assess factors associated with patient and graft survival. Renal graft loss was defined as return to dialysis, new transplant, or death with a functioning graft. The SPSS® program version 28 was used for analysis.

## Results

Data from 99 KT due to LN performed from January 1977 to June 2023, with follow-up until December 2023, were analyzed.

### Clinical and Demographic Characteristics

Baseline demographic, clinical, and immunological characteristics are shown in Table 1, considering the two different immunosuppression periods (G1, before 2009, and G2, from 2009 onwards). The median age was significantly lower in G1 compared to G2, both at SLE diagnosis [16.0 (8.5–21.0) *vs.* 19.0 (12.0–26.0) years, p = 0.016] and at kidney transplantation [24.5 (20.0–33.2) *vs.* 31.0 (24.5–44.0) years, p = 0.002]. The groups were similar in their distribution by sex (86.9% female), comorbidities, lupus-related autoantibodies, and HLA-DR typing. In the second group, there was a higher proportion of non-white patients compared to G1 (p = 0.025). The histological class of LN was duly documented in the medical records of 35 (35.3%) patients: 2 (2.0%) class III, 26 (26.2%) class IV, 3 (3.0%) class V, and 4 (4.0%) class VI. Regarding SLE treatment prior to KT, there was a predominance in the use of azathioprine (56.6% *vs.* 30.4%, p = 0.045) for G1, and mycophenolate for G2 (3.3% *vs.* 33.3%, p < 0.001).

**Table 1:** Clinical and demographic data on 99 kidney transplant recipients due to lupus nephritis

Characteristica	N = 99	G1 (KT until 2009) N = 30	G2 (KT from 2009 onwards) N = 69	P
**Age, years (median, P 25–75)** At SLE diagnosis At kidney transplantation	20.0 (11.5–25.0) 30.0 (23.0–40.0)	16.0 (8.5–21.0) 24.5 (20.0–33.2)	19.0 (12.0–26.0) 31.0 (24.5–44.0)	**0.016** **0.002**
Female sex (%)	86 (86.9)	26 (86.6)	60 (86.9)	0.96
**Ethnicity (%)** White Brown Black Indigenous	66 (66.7) 22 (22.2) 10 (10.1) 1 (1.0)	25 (83.3) 3 (10.0) 2 (6.6) 0	41 (59.5) 19 (27.5) 8 (11.6) 1 (1.4)	**0.025**
**Pre–KT comorbidities (%)** Systemic arterial hypertension Viral hepatitis Previous neoplasm Previous tuberculosis Diabetes *mellitus*	76 (76.7) 16 (15.1) 5 (4.0) 4 (4.0) 3 (3.0)	20 (66.6) 6 (20.0) 1 (3.3) 0 1 (3.3)	58 (84.0) 10 (14.5) 4 (5.8) 4 (5.8) 2 (2.9)	0.33 0.19 1.00 0.57 1.00
Presence of SLE–related autoantibodies* (%) Antinuclear factor Anti–double strand DNA Lupus anticoagulant Anti–cardiolipin	55 (54.5) 29 (29.3) 20 (20.2) 13 (13.1)	15 (50.0) 5 (16.6) 3 (10.0) 1 (3.3)	40 (57.9) 24 (34.7) 17 (24.6) 12 (17.4)	0.606 0.297 0.731 0.426
Decreased serum complement* (%)	31 (31.3)	5 (16.6)	26 (37.6)	0.189
**HLA–DR typing (%)** DR3 DR4 DR11 DR15	5 (5.1) 23 (23.2) 14 (14.1) 23 (23.2)	1 (3.3) 10 (33.3) 3 (10.0) 6 (20.0)	4 (5.8) 13 (18.8) 11 (15.9) 17 (24.6)	1.00 0.60 0.75 1.00
**Histological class of lupus nephritis (%)** Class III Class IV Class V Class VI Not Available	2 (2.0) 26 (26.2) 3 (3.0) 4 (4.0) 64 (64.6)	1 (3.3) 2 (6.6) 1 (3.3) 0 26 (86.6)	1 (1.4) 24 (34.7) 2 (2.9) 4 (5.8) 38 (55.0)	**0.020**
**SLE treatment prior to transplantation (%)** Hydroxychloroquine Cyclophosphamide Azathioprine Mycophenolate Rituximab Calcineurin inhibitors	48 (48.4) 31 (31.3) 38 (38.3) 24 (24.2) 6 (6.0) 4 (4.0)	9 (30.0) 6 (16.6) 17 (56.6) 1 (3.3) 0 0	39 (56.5) 25 (36.2) 21 (30.4) 23 (33.3) 6 (8.7) 4 (5.8)	**0.003** 0.063 **0.045** **< 0.001** 0.171 0.302

Abbreviations – SLE: systemic lupus erythematosus; KT: kidney transplantation. Notes – *presence of SLE-related autoantibodies or reduced complement at any time prior to kidney transplantation.

### Characteristics and Events Related to Kidney Transplantation

Patients were divided into two groups, defined by the difference in immunosuppression protocol. The proportions are detailed in Table 2. Within G1, patients predominantly received cyclosporine with azathioprine, while in G2 they received tacrolimus and mycophenolate. Induction therapy with thymoglobulin was started in 2009.

**Table 2:** Events related to kidney transplantation in 99 patients with lupus nephritis

	N = 99	G1 (KT until 2009) N = 30	G2 (KT from 2009 onwards) N = 69	P
Time from SLE to KT, years (median, P25–75)	9.7 (2.8–16.1)	3.7 (0.7–10.5)	10.0 (2.8–16.1)	0.104
Dialysis vintage until KT, years (median, P25–75)	1.5 (0.6–3.5)	1.0 (0.3–2.1)	1.7 (0.6–3.8)	0.160
Follow–up after KT, years (median, P25–75)	5.0 (1.7–10.4)	14.0 (7.7–17.2)	3.0 (1.0–6.5)	**< 0.001**
**Type of dialysis** Hemodialysis Peritoneal Both Preemptive	79 (79.8) 5 (5.0) 10 (10.1) 5 (5.0)	24 (80.0) 2 (6.6) 1 (3.3) 3 (10.0)	55 (79.7) 3 (4.3) 9 (13.0) 2 (2.9)	0.253
Living donor (%)	28 (28.2)	15 (50.0)	13 (18.8)	**0.003**
**First kidney transplantation (%)**	83 (83.8)	29 (96.6)	54 (78.2)	**0.035**
**Panel reactivity** Median (P25–75) ≥ 80%	26.0 (2.8–60.0) 11 (11.1)	6.5 (0–17.0) 0	26.0 (3.5–65.5) 11 (15.9)	**0.039** 0.583
**Donor anti–HLA antibody** Yes 1 2	11 (11.1) 7 (7.1) 4 (4.0)	0 0 0	11 (15.9) 7 (10.1) 4 (5.8)	1.000
**Immunosuppression (%)** Thymoglobulin Basiliximab Cyclosporine Tacrolimus Azathioprine Mycophenolate	40 (40.4) 30 (30.3) 25 (25.2) 73 (73.7) 20 (20.2) 79 (79.8)	0 4 (13.3) 23 (76.6) 6 (20.0) 18 (60.0) 12 (40.0)	40 (57.9) 26 (37.6) 2 (2.9) 67 (97.1) 2 (2.9) 67 (97.1)	**< 0.001** **0.018** **< 0.001** **< 0.001** **< 0.001** **< 0.001**
**Post–transplant complications (%)** Bacterial infections Viral infections Neoplasm Bleeding Cardiological	49 (49.4) 27 (27.2) 12 (12.1) 7 (7.0) 6 (6.0)	10 (33.3) 3 (10.0) 6 (20.0) 0 5 (16.6)	39 (56.5) 24 (34.7) 6 (8.7) 7 (10.1) 1 (1.4)	0.359 0.519 **0.006** 0.589 **< 0.001**
Glomerular filtration rate* (median, P25–75) 1 year 5 years	60.0 (46.0–74.1) 49.5 (31.8–65.8)	64.5 (48.7–71.5) 50.0 (37.7–66.3)	59.7 (42.7–76.2) 45.6 (30.9–63.2)	0.576 0.331
**Proteinuria/creatininuria ratio (median, P25–75)** 1 year 5 years	0.20 (0.10–0.40) 0.27 (0.15–0.66)	0.10 (0.10–0.30) 0.23 (0.10–0.50)	0.20 (0.15–0.50) 0.36 (0.15–0.79)	0.089 0.350
**Renal graft biopsy** Yes Rejection Lupus recurrence	46 (46.4) 23 (23.2) 5 (5.0)	15 (50.0) 5 (16.6) 4 (13.3)	31 (44.9) 18 (26.0) 1 (1.4)	0.504 0.301 **0.033**
**Post–kidney transplantation follow–up (%)** Outpatient follow–up Graft loss Death with functioning graft Loss to follow–up	45 (45.5) 37 (37.4) 13 (13.1) 4 (4.0)	5 (16.6) 20 (66.7) 5 (16.6) 0	40 (58.0) 17 (24.6) 8 (11.6) 4 (5.8)	**< 0.001**
**Cause of graft loss** Vascular thrombosis Rejection Infection	6 (6.0) 25 (25.2) 6 (6.0)	0 19 (63.3) 1 (3.3)	6 (8.7) 6 (8.7) 5 (7.2)	**0.009**
**Cause of death** Cardiovascular Infection Neoplasm Others	4 (4.0) 5 (5.0) 2 (2.0) 2 (2.0)	1 (3.3) 3 (10.0) 1 (3.3) 0	3 (4.3) 2 (2.9) 1 (1.4) 2 (2.9)	0.375

Abbreviations – SLE: systemic lupus erythematosus; KT: kidney transplantation. Notes – *CKD-EPI 2021 equation based on creatinine.

The median time between SLE diagnosis and KT was 9.75 (2.83–16.12) years, similar between G1 and G2. The median time from initiation of dialysis to KT was 1.5 (0.6–3.5) years, similar between the groups. Patients in G1 more frequently received a first transplant (96.6% *vs.* 78.2%, p = 0.035) from a living donor (50% *vs.* 18.8%, p = 0.003). Median panel reactive antibody was lower in G1 compared to G2 [6.5 (0–17.0) *vs.* 26.0 (3.5–65.5), p = 0.039].

### Extrarenal Manifestations of SLE

Regarding the extrarenal manifestations of SLE, there was a significant reduction in patients with joint complaints [71.8% *vs.* 52.5%, OR 0.70 (0.010–0.472), p < 0.001], mucocutaneous manifestations (photosensitivity and malar rash) [70.6% *vs.* 52.5%, OR 0.026 (0.004–0.183), p < 0.001], and neurological manifestations (seizures) [12.1% *vs.* 8.1%, OR 0.046 (0.014–0.153), p < 0.001] before and after KT, respectively. There was no difference in the frequency of thrombotic, obstetric, ophthalmologic events, or serositis (not shown). The presence of lupus anticoagulant, anticardiolipin antibody, or anti-beta 2 glycoprotein 1 antibody, whether isolated or in combination, was not associated with pre-transplant thrombosis, post-transplant thrombosis, or post-transplant bleeding (not shown).

### Post-Transplant Follow-Up

The main post-transplant complications were bacterial (48.4%) and viral (26.2%) infections, which were similar between groups. The development of neoplasms was significantly higher in G1 compared to G2 (20% *vs.* 8.7%, p = 0.006, respectively), with emphasis on those related to HPV (n = 5), breast (n = 3), lymphoma (n = 2), and kidney (n = 1). Cardiovascular disease was also significantly more frequent in G1 compared to G2 (16.6% *vs.* 1.4%, p < 0.001).

GFR at 1 year (64.5 *vs.* 59.7 mL/min/1.73m^2^, p = 0.57) and at 5 years (50.0 *vs.* 45.6 mL/min/1.73m^2^, p = 0.33), as well as proteinuria at 1 year (0.10 *vs.* 0.20, p = 0.08), and at 5 years (0.23 *vs.* 0.36, p = 0.35) were similar between both groups.

Renal graft biopsy was indicated in 46% of patients, with a similar proportion of rejection episodes in both groups (16.6% *vs.* 26.0%, p = 0.30). There was recurrence of LN after transplantation in 4 (13.3%) patients in G1, and in 1 (1.4%) patient in G2, p = 0.033. The median time to recurrence was 9.0 (5.5–10.0) years, similar between the groups (p = 0.051). The 4 patients in G1 with recurrence lost their graft function and returned to dialysis at a median of 2.5 (0.5–6.7) years, with subsequent biopsies revealing rejection. The G2 patient with LN recurrence is currently under follow-up (5 years after recurrence), with a GFR of 45 mL/min/1.73m^2^, and a PCR of 0.1. No other factors were found to be significantly associated with recurrence, except for the transplantation period: age, sex, skin color, presence of pre-transplant autoantibodies, HLA-DR typing, cPRA, immunosuppression, donor type, and rejection.

### Patient and Kidney Graft Survival

The median follow-up time after KT was 5.0 (1.7–10.4) years, which was longer in G1 compared to G2 (14.0 *vs.* 3.0, p < 0.001), as expected once this was the oldest cohort. Graft loss occurred in 37 (37.4%) patients, and death with a functioning graft in 13 (13.1%) during the entire follow-up period. The causes of renal graft loss included vascular thrombosis (n = 6), infection (n = 6), and rejection (n = 25). No graft loss was attributed to LN recurrence. Among the causes of death with a functioning graft, 4 were cardiovascular, 2 due to neoplasms, and 5 caused by infection, 4 of which resulting from SarsCov2 infection.

**Figure 1:**
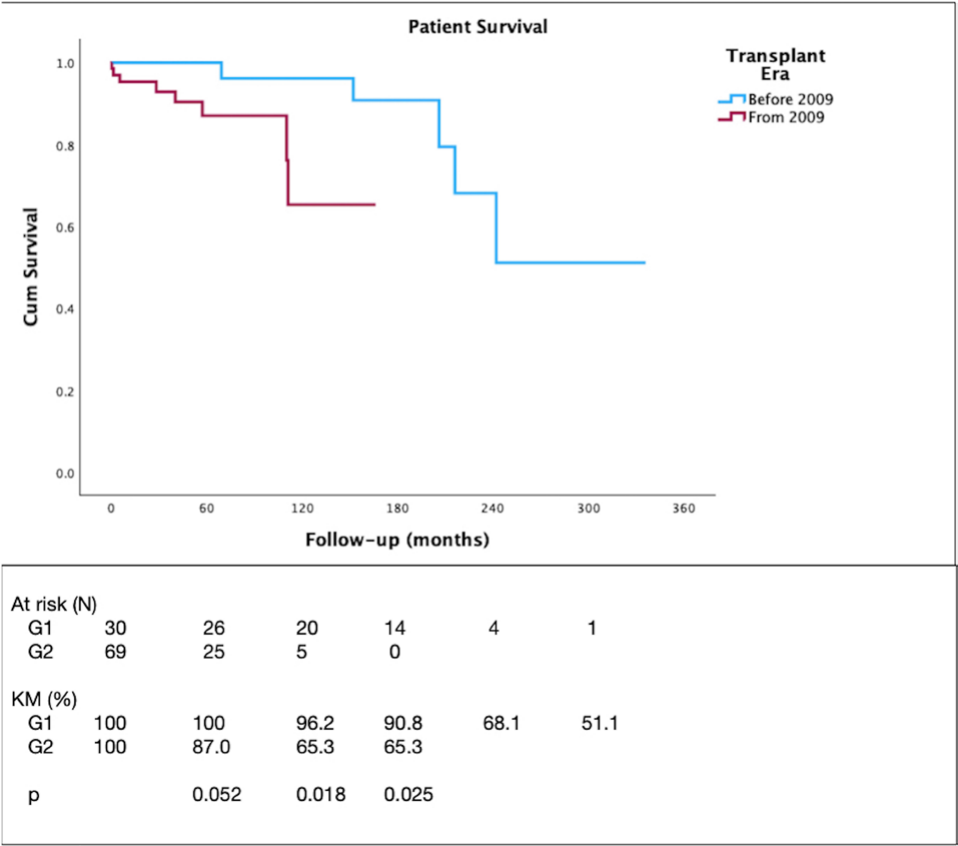
Patient survival after kidney transplantation for lupus nephritis.

**Figure 2:**
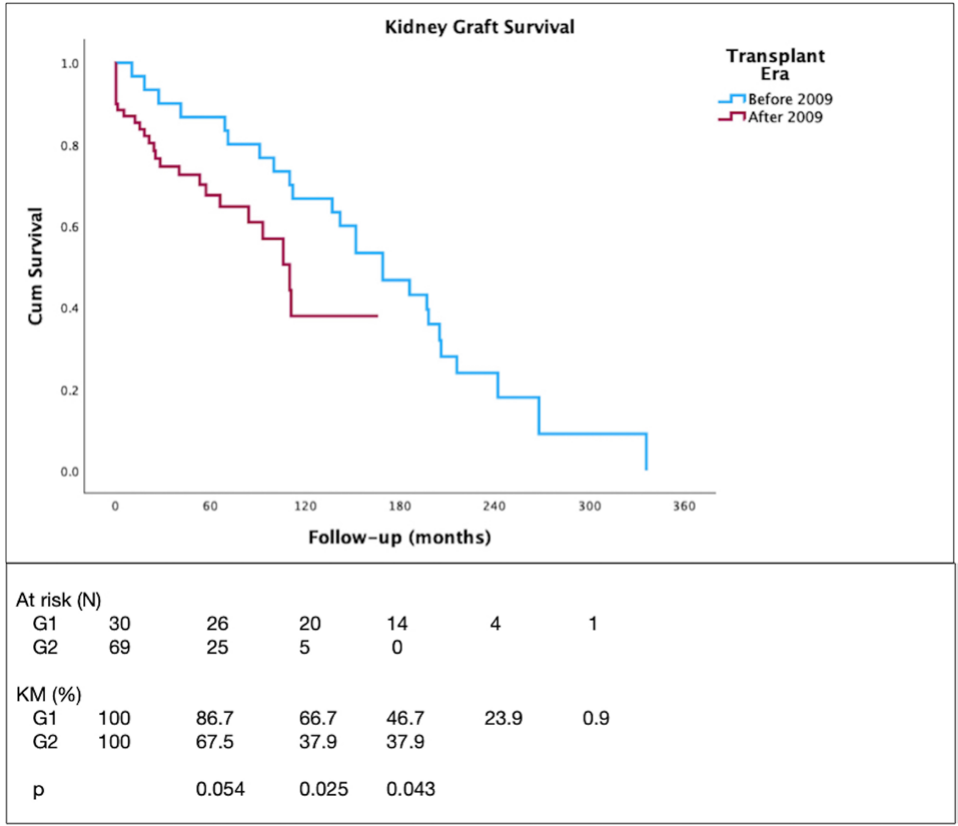
Graft survival after kidney transplantation for lupus nephritis.

The 5-year graft survival was 73.6%, similar to that of patients transplanted due to other causes (71.5%, p = 0.308) (not shown). The patient and graft survival curves are depicted in Figures 1 and 2, respectively. There were no deaths within 5 years in G1, while patient survival in G2 was 87%. The 5-year graft survival was similar between the groups (86.7% *vs.* 67.5%, p = 0.054). Patient and graft survival rates were significantly higher in G1 compared to G2 at 10 years (96.2% *vs.* 65.3%, p = 0.018, and 66.7% *vs.* 37.9%, p = 0.025, respectively), and at total follow-up period (51.1% *vs.* 65.3%, p = 0.025, and 53.3% *vs.* 37.9%, p = 0.043, respectively). In Cox regression analysis, no factors were identified as being associated with graft loss or death at 5 years, 10 years, or throughout follow-up. The included variables were sex, age at transplantation, type of dialysis, type of donor, cPRA, presence of anti-HLA antibody, HLA-DR typing, rejection episodes, LN recurrence, GFR at 1 year, and PCR at 1 year.

## Discussion

This study was conducted at a single center, assessing the evolution, complications, and survival of 99 patients who underwent kidney transplantation due to lupus nephritis (LN) between 1977 and 2023. Two groups were compared, divided by the change in immunosuppression: prior to 2009 (G1), and from 2009 onwards (G2), with comparable graft function and survival at 5 years. The indication for renal graft biopsy was similar in both groups, with a higher recurrence of LN in G1. LN recurrence documented by biopsy was not associated with graft loss.

Prevalence studies place the diagnosis of LN in women between the third and fourth decades of life, with higher frequency among African-Americans and Afro-Caribbeans, and greater severity of kidney disease among Black and Hispanic individuals^1,3^. Our study population was mostly comprised of white women diagnosed with SLE between the second and third decades of life, who underwent KT between the third and fourth decades of life. This ethnic difference reflects specific characteristics of our local population^9^.

The primary comorbidity was systemic arterial hypertension, similar to the findings from other studies^8,10^. Hypertension is associated with the severity of kidney injury and the type of histological lesion, as well as with immune dysregulation and activation of the renin-angiotensin-aldosterone system, corticosteroid use, and accelerated atherosclerosis due to chronic inflammation^10^.

Several clinical and demographic factors have been investigated and associated with the progression of LN before and after kidney transplantation. Male sex has been reported as a higher risk factor for LN progression^3,11^, and in the present study it was not associated with the time between SLE diagnosis and KT, nor did it influence survival. The HLA-DR3 and HLA-DR15 typing were associated with unfavorable LN presentation^3,5^; however, there was no association with graft survival^12^. The HLA-DR4 and DR11 serotypes are described as protective factors against the development of LN^3,5,13^. In our study, we found a significant frequency of these alleles, but no direct association with the clinical outcome was identified.

Renal replacement therapy modality (hemodialysis *versus* peritoneal dialysis) had no impact on survival, in line with a 2020 review, comparable to that of patients undergoing dialysis due to other causes^3^. KT provides better quality of life and increased survival compared to dialysis in various contexts^14,15,16^, including LN^3^. It results in reduced all-cause mortality and mortality from cardiovascular disease, coronary artery disease, infection, and sepsis compared to non-transplanted patients^8^.

The main extrarenal manifestations of SLE tend to disappear in dialysis patients, including the negativity of immune activity markers, even allowing immunosuppression to be withdrawn^17^. However, a recent study by Kim et al. observed that up to one-quarter of LN patients on dialysis experienced exacerbations of SLE, mainly hematological and constitutional manifestations^18^. In our study, approximately half of the patients exhibited negative immunological markers during the assessment for KT. The primary extrarenal manifestations of SLE were mucocutaneous, articular, and hematological, all of which significantly decreased in frequency after KT.

The risk of developing kidney failure in LN depends on several factors, including histological class, healthcare access, and response to immunosuppressive treatments^3,19^. Proteinuria control is the main parameter for disease control and for defining partial or complete response, although there is no consensus on target values^3^. A systematic review and meta-analysis published in 2016 identified a reduction in the risk of progression to kidney failure over the decades, possibly related to the development of immunosuppression protocols; however, this has stagnated in recent years. The risk in developed countries is 10%, 15%, and 20% at 5, 10 and 15 years, respectively, being up to 10% higher in developing countries^6^. The evolution in the treatment of LN was demonstrated in this study, with a predominance of azathioprine use in the early decades, transitioning to mycophenolate in more recent years, along with the introduction of calcineurin inhibitors and rituximab.

Class IV LN is associated with a higher risk of progression to kidney failure, reaching 44% within 15 years, a figure twice as high as the risk in class V LN^6^. Therefore, identifying the histological class is important in the risk stratification and treatment of LN. Conversely, for patients progressing to kidney disease stage V, there is no benefit in performing a renal biopsy, as the histopathological findings will not change the management regarding renal replacement treatment^20^. In the present study, documentation of histological class was limited, with most patients presenting with class IV, as expected for patients with end-stage renal disease. We could attribute this lack of records to the fact that patients were referred for KT while already undergoing dialysis treatment, after being followed up and treated at other centers, possibly neglecting the importance of this information in the context of kidney failure.

The median times between the diagnosis of SLE and kidney transplantation, as well as from the initiation of dialysis to transplantation, were similar in groups G1 and G2. However, G1 more frequently received living donor transplants (50% *vs.* 18.8%), consistent with the findings from the Brazilian Transplant Registry over time^21^. The type of donor has an impact on patient and graft survival^21^, and on post-transplant complications, but no such association was identified in the present study.

One of the factors related to early graft loss is the presence of antiphospholipid antibodies^3^, which is frequent in SLE patients, although the development of antiphospholipid syndrome (APS) is less common. Ames et al. demonstrated that patients with antiphospholipid antibodies are at greater risk of thrombotic events, including thrombotic microangiopathy in the graft, and should ideally be screened prior to transplantation^22^. The APS screening workup is expensive, and at our center it is performed on patients with identifiable risk factors in addition to the diagnosis of SLE, such as previous thrombosis events or miscarriages. The frequency of antiphospholipid antibody positivity in our study was low; no statistically significant association was found between the presence of these antibodies and post-transplant thrombosis or graft loss. Possibly there was no statistical power to demonstrate this association. However, we should also emphasize that in patients identified as being at greater risk of thrombosis, perioperative anticoagulation is initiated per protocol.

The pro-inflammatory state related to SLE, heart valve disease, the presence of antiphospholipid antibodies, and previous immunosuppressive treatments may increase the risk of infectious, cardiac, and cerebrovascular complications following kidney transplantation^4,23^. Prolonged pre- and post-KT corticosteroid therapy could aggravate cardiovascular disease and predispose patients to osteoporosis, diabetes *mellitus*, cataracts, and myopathies, among others. The frequency and severity of infections also seem to be higher in SLE patients, especially in those who received prolonged immunosuppression prior to KT^24^. In this study, the main complications observed were bacterial and viral infections, followed by neoplasms and, less frequently, cardiac complications.

The risk of developing malignant neoplasms is associated with the use of immunosuppressants prior to transplantation in general^25,26^. In addition, SLE patients have an increased risk of neoplasms compared to the general population, regardless of KT^4,27,28^. However, studies have shown that LN patients who have received kidney transplants appear to have the same risk of developing malignancies (excluding melanoma) as patients who received kidney transplants due to other causes^29^. In the present study, the frequency of neoplasms was higher than that found in another survey conducted at the institution^30^, most of which related to HPV.

Renal function at 1 year and at 5 years was similar between the two groups, with different immunosuppression.

Post-KT recurrence of LN is estimated at 2% to 11%^3,31,32,33^; potentially reaching over 50% in protocol biopsies^34^. The most common presentation is class II, with no impact on graft survival^3,32,34^. In our sample, the recurrence of LN was 5%, and the demonstration of classes IV, V, and VI is justified by the fact that biopsies were indicated due to graft dysfunction or proteinuria rather than per protocol. The median time to recurrence was 9 years, with no graft loss attributed to LN recurrence. There was a higher frequency of recurrence in the group with longer follow-up period, but no association with immunosuppression was identified.

Graft loss occurred in 37.4% of patients, primarily due to rejection and infection. Death with a functioning graft occurred in 13% of patients, mainly due to cardiovascular causes, infection, and neoplasms.

There are discrepancies as to graft survival in patients with LN compared to KT due to other causes, with studies showing inferior results^35,36,37,38,39^, while others report comparable^40,41,42,43,44^, and even superior survival rates^12^. Our study showed a 5-year graft survival comparable to those transplanted for other causes, and to the institution’s historical KT records^45,46^ in different scenarios. However, at 10 years and throughout follow-up, patient and kidney graft survival rates were higher in G1 compared to G2. No evaluated variables were significantly associated with this difference, namely sex, age at transplant, type of dialysis, donor type, cPRA, presence of anti-HLA antibody, HLA-DR typing, rejection episodes, LN recurrence, GFR at 1 year, and proteinuria at 1 year. Of note, the number of G2 patients who reached a follow-up period of 10 years or more was very small, which may explain the difference in survival rates.

The main limitations of the study are related to its retrospective nature, covering an extended period, which may have compromised the accuracy of some clinical information, especially before the implementation of electronic medical records. Among the positive aspects of the study, it is worth noting that the large number of kidney transplant patients due to LN at the institution and the long follow-up period provide valuable data on long-term outcomes.

In conclusion, LN is a serious manifestation of SLE, leading to kidney failure. Kidney transplantation is a successful treatment for these patients, with graft survival rates similar to those of patients transplanted due to other causes. LN recurrence was not associated with graft loss.
