# Supplementary material for: Epidemiological profile of kidney transplant patients with lupus nephritis
Source: J Bras Nefrol. 2024 Dec 13;47(2):e20240061. doi: 10.1590/2175-8239-JBN-2024-0061en (PMC11642653; doi:10.1590/2175-8239-JBN-2024-0061en)
Supplement: Translation

# Translation: Perfil epidemiológico de pacientes transplantados renais portadores de nefrite lúpica

**DOI:** 10.1590/2175-8239-JBN-2024-0061pt

**Authors:** Beatriz Curto Pachi, Laura Marcon Bischoff Bialecki, Luísa Rigon Borba, Helena Marcon Bischoff, Valter Duro Garcia, Gisele Meinerz, Elizete Keitel

**Affiliations:** 1 Santa Casa de Porto Alegre, Porto Alegre, Rio Grande do Sul, Brazil.; 2 Universidade Federal de Ciências da Saúde de Porto Alegre, Porto Alegre, Rio Grande do Sul, Brazil.

**Keywords:** Lupus Eritematoso Sistêmico, Nefrite Lúpica, Transplante Renal

## Abstract

**Introdução:**

A nefrite lúpica (NL) afeta até 50% dos pacientes com lúpus eritematoso sistêmico (LES) e pode levar à falência renal e necessidade de transplante renal (TR). Os resultados em comparação à TR por outras causas são controversos, e visamos avaliar evolução clínica, complicações e sobrevida de pacientes com NL submetidos a TR.

**Metodologia:**

Coorte retrospectiva de 99 TR por NL de 1977 a 2023 em um único centro, divididos em dois grupos conforme o período de imunossupressão: G1 (antes de 2009) e G2 (a partir de 2009). As características clínicas, demográficas e evolução clínica foram comparadas.

**Resultados:**

Pacientes eram na maioria brancos (65,9%), sexo feminino (86,9%), no primeiro TR (83,8%). A idade mediana foi 20,0 (11,5–25,0) anos no diagnóstico de LES e 30,0 (23,0–40,0) anos no TR. Biópsia do enxerto renal foi indicada para 46% dos pacientes, com rejeição em 23% e recorrência de NL em 5%. Avaliando os dois períodos distintos de imunossupressão padrão, não houve diferença na mediana de taxa de filtração glomerular e de proteinúria em 1 ano e em 5 anos, nem da sobrevida em 5 anos. Em todo o acompanhamento, 37,4% dos pacientes perderam o enxerto e 13% morreram com enxerto funcionante. Nenhuma perda foi atribuída à recorrência de NL.

**Conclusão:**

TR é um tratamento bem-sucedido na NL, com taxas de sobrevida do enxerto semelhantes às de transplantes por outras causas. A recorrência de NL não esteve associada à perda do enxerto renal.

## Introdução

O lúpus eritematoso sistêmico (LES) é uma doença inflamatória crônica sistêmica de origem autoimune, com apresentações clínicas heterogêneas e curso de remissão e recorrência. Há uma grande variabilidade na incidência/prevalência dessa doença ao redor do mundo^1^. Um estudo brasileiro estimou a incidência em 8,7 casos a cada 100.000 habitantes^2^.

A nefrite lúpica (NL) é a causa mais frequente de lesão renal no LES, ocorrendo em 20% a 60% dos pacientes^3,4^. Alguns fatores parecem estar associados a pior prognóstico renal, como etnias negra e hispânica comparadas à branca, sexo masculino e sorotipos HLA-DR3 e DR15. Os sorotipos HLA-DR4 e DR11 parecem ter papel protetor renal^3,5^.

Indivíduos com NL têm maior mortalidade comparados a pacientes sem acometimento renal, chegando a resultados entre 5% a 25% em 5 anos a partir do início da doença^6^. Em 10 anos, mais de 50% dos pacientes que respondem parcialmente ao tratamento e mais de 85% dos que não respondem podem necessitar de terapia renal substitutiva^7^. Esses pacientes são candidatos a transplante renal (TR), podendo apresentar boa função do enxerto, baixa recorrência (2% a 11%)^3^ e melhor sobrevida^8^, com menos eventos cardiovasculares e menos infecções que os pacientes com NL que permanecem em diálise^3^. De interesse especial, pacientes com histórico de trombose ou abortos devem ser avaliados quanto à possibilidade de síndrome antifosfolipídeo antes do transplante, para prevenir complicações periprocedimento^3^.

O presente estudo busca traçar o perfil epidemiológico dos pacientes com NL transplantados na Santa Casa de Porto Alegre, avaliando suas características, evolução clínica e sobrevida do enxerto após o transplante renal. A revisão de literatura é controversa em termos de resultados em comparação a transplantes por outras causas, e visamos avaliar os fatores associados a desfechos em longo prazo na nossa população.

## Métodos

### Desenho

Estudo observacional de coorte retrospectiva.

### Amostra

Pacientes que realizaram transplante renal por NL no período de janeiro de 1977 a junho de 2023, com acompanhamento até dezembro de 2023. O critério de exclusão foi a realização de transplante concomitante de outro órgão. Se o mesmo paciente foi submetido a mais de um transplante renal no período, foi contabilizado como um caso para cada procedimento.

### Comitê de Ética

O projeto foi aprovado pelo comitê de ética em pesquisa com seres humanos da Instituição (protocolo número 70505123.5.3001.5345).

### Coleta de Dados

Os receptores de TR são acompanhados pela equipe, e suas informações demográficas, características clínicas e imunológicas, características do doador e status de acompanhamento são mantidos atualizados em um banco de dados pelo Serviço de Nefrologia, usado para identificar os pacientes com NL. Foram considerados pacientes com NL aqueles referenciados para realizar o TR com esse diagnóstico, sem evidência de outra causa para a falência renal.

### Variáveis

Foram pesquisadas características clínicas, demográficas e laboratoriais dos receptores e dados dos doadores, terapia imunossupressora, complicações perioperatórias, cardiovasculares, infecciosas e desenvolvimento de malignidades, sobrevida do enxerto e do paciente. A taxa de filtração glomerular foi calculada usando equação baseada em creatinina sérica CKD-EPI 2021, em mL/min/1,73m^2^. A proteinúria foi calculada como relação proteinúria/creatininúria (RPC) em amostra de urina. As manifestações extrarrenais do LES foram levantadas de acordo com os registros em prontuário.

### Acompanhamento

Os pacientes são acompanhados ambulatorialmente após o TR, mensalmente no primeiro ano e depois bimensalmente, enquanto houver função do enxerto renal. Em todas as consultas são coletados exames de função renal e de urina. Biópsias renais são realizadas por indicação clínica, por exemplo, piora de função renal e/ou proteinúria, e não de forma protocolar. As biópsias de enxerto renal são analisadas no laboratório de patologia do hospital.

### Imunossupressão

Os protocolos de imunossupressão foram modificados ao longo do período de avaliação do estudo. Os primeiros anos foram baseados em azatioprina e prednisona, com a introdução de ciclosporina a partir da década de 1990. A terapia de indução começou a ser mais utilizada a partir dos anos 2000, baseada principalmente em basiliximabe. A partir de 2009, o protocolo principal foi baseado em tacrolimus, micofenolato e prednisona, e a terapia de indução em basiliximabe ou timoglobulina, para pacientes de maior risco imunológico. Dessa forma, consideramos dois períodos de imunossupressão: G1, antes de 2009, baseado em ciclosporina, azatioprina e prednisona, e G2, a partir de 2009, baseada em tacrolimus, micofenolato e prednisona, com maior uso de terapia de indução.

### Análise Estatística

As variáveis categóricas foram descritas como frequências absolutas (n) e percentuais (%) e comparadas por qui-quadrado e teste exato de Fisher. As variáveis contínuas foram apresentadas como medidas de tendência central e dispersão e comparadas com testes paramétricos e não paramétricos de acordo com a distribuição normal. O nível de significância de 5% (p < 0,05) foi considerado para todos os testes estatísticos. A sobrevida do paciente e do enxerto renal foram avaliadas por Kaplan-Meier, e a significância determinada por teste de log-rank. Regressão de Cox foi utilizada para avaliar fatores associados à sobrevida do paciente e do enxerto. A perda do enxerto renal foi definida como retorno à diálise, novo transplante ou óbito com enxerto funcionante. Para análise, foi utilizado o programa SPSS® versão 28.

## Resultados

Foram analisados dados de 99 TR por NL no período de janeiro de 1977 a junho de 2023, com acompanhamento até dezembro de 2023.

### Características Clínicas e Demográficas

As características demográficas, clínicas e imunológicas basais estão apresentados na Tabela 1, considerando os dois diferentes períodos de imunossupressão (G1, antes de 2009, e G2, a partir de 2009). A mediana de idade foi significativamente menor no G1 comparado com o G2, tanto ao diagnóstico de LES [16,0 (8,5–21,0) *vs*. 19,0 (12,0–26,0) anos, p = 0,016] quanto ao transplante renal [24,5 (20,0–33,2) *vs*. 31,0 (24,5–44,0) anos, p = 0,002]. Os grupos foram semelhantes na distribuição por sexo (feminino em 86,9%), comorbidades, autoanticorpos relacionados ao lúpus e tipagem HLA-DR. No segundo grupo, houve maior proporção de pacientes não brancos comparados ao G1, p = 0,025. A classe histológica da NL foi devidamente documentada no prontuário de 35 (35,3%) pacientes: 2 (2,0%) classe III, 26 (26,2%) classe IV, 3 (3,0%) classe V e 4 (4,0%) classe VI. Quanto ao tratamento do LES antes do TR, houve predomínio de uso de azatioprina (56,6% *vs*. 30,4%, p = 0,045) no G1 e de micofenolato no G2 (3,3 % *vs*. 33,3%, p < 0,001).

**Tabela 1:** Dados clínicos e demográficos de 99 receptores de transplante renal por nefrite lúpica

Característica	N = 99	G1 (TR até 2009) N = 30	G2 (TR a partir 2009) N = 69	P
**Idade, anos (mediana, P 25–75)** Ao diagnóstico de LES Ao transplante renal	20,0 (11,5–25,0) 30,0 (23,0–40,0)	16,0 (8,5–21,0) 24,5 (20,0–33,2)	19,0 (12,0–26,0) 31,0 (24,5–44,0)	**0,016** **0,002**
Sexo feminino (%)	86 (86,9)	26 (86,6)	60 (86,9)	0,96
**Cor de pele (%)** Branca Parda Preta Indígena	66 (66,7) 22 (22,2) 10 (10,1) 1 (1,0)	25 (83,3) 3 10,0) 2 (6,6) 0	41 (59,5) 19 (27,5) 8 (11,6) 1 (1,4)	**0,025**
**Comorbidades pré–TR (%)** Hipertensão arterial sistêmica Hepatite viral Neoplasia prévia Tuberculose prévia Diabetes *mellitus*	76 (76,7) 16 (15,1) 5 (4,0) 4 (4,0) 3 (3,0)	20 (66,6) 6 (20,0) 1 (3,3) 0 1 (3,3)	58 (84,0) 10 (14,5) 4 (5,8) 4 (5,8) 2 (2,9)	0,33 0,19 1,00 0,57 1,00
Presença de autoanticorpos relacionados ao LES* (%) Fator antinuclear Anti–DNA dupla hélice Anti–coagulante lúpico Anti–cardiolipina	55 (54,5) 29 (29,3) 20 (20,2) 13 (13,1)	15 (50,0) 5 (16,6) 3 (10,0) 1 (3,3)	40 (57,9) 24 (34,7) 17 (24,6) 12 (17,4)	0,606 0,297 0,731 0,426
Diminuição de complemento sérico* (%)	31 (31,3)	5 (16,6)	26 (37,6)	0,189
**Tipagem HLA–DR (%)** DR3 DR4 DR11 DR15	5 (5,1) 23 (23,2) 14 (14,1) 23 (23,2)	1 (3,3) 10 (33,3) 3 (10,0) 6 (20,0)	4 (5,8) 13 (18,8) 11 (15,9) 17 (24,6)	1,00 0,60 0,75 1,00
**Classe histológica da nefrite lúpica (%)** Classe III Classe IV Classe V Classe VI Indisponível	2 (2,0) 26 (26,2) 3 (3,0) 4 (4,0) 64 (64,6)	1 (3,3) 2 (6,6) 1 (3,3) 0 26 (86,6)	1 (1,4) 24 (34,7) 2 (2,9) 4 (5,8) 38 (55,0)	**0,020**
**Tratamento do LES prévio ao transplante (%)** Hidroxicloroquina Ciclofosfamida Azatioprina Micofenolato Rituximabe Inibidores da calcineurina	48 (48,4) 31 (31,3) 38 (38,3) 24 (24,2) 6 (6,0) 4 (4,0)	9 (30,0) 6 (16,6) 17 (56,6) 1 (3,3) 0 0	39 (56,5) 25 (36,2) 21 (30,4) 23 (33,3) 6 (8,7) 4 (5,8)	**0,003** 0,063 **0,045** **< 0,001** 0,171 0,302

Abreviaturas – LES: lúpus eritematoso sistêmico; TR: transplante renal. Nota – *presença de autoanticorpos relacionados ao LES ou de complemento reduzido em qualquer período pré-transplante renal

### Características e eventos relacionados ao transplante renal

Os pacientes foram divididos em dois grupos, definidos pela diferença no protocolo de imunossupressão, e as proporções estão detalhadas na Tabela 2. No G1, os pacientes receberam majoritariamente ciclosporina com azatioprina, e no G2, tacrolimus e micofenolato. A terapia com indução com timoglobulina foi iniciada a partir de 2009.

**Tabela 4:** Eventos relac ionados ao transplante renal em 99 pac ientes com nefrite lúpica

	N = 99	G1 (TR até 2009) N = 30	G2 (TR a partir 2009) N = 69	P
Tempo de LES até TR, anos (mediana, P25–75)	9,7 (2,8–16,1)	3,7 (0,7–10,5)	10,0 (2,8–16,1)	0,104
Tempo de diálise até TR, anos (mediana, P25–75)	1,5 (0,6–3,5)	1,0 (0,3–2,1)	1,7 (0,6–3,8)	0,160
Acompanhamento após TR, anos (mediana, P25–75)	5,0 (1,7–10,4)	14,0 (7,7–17,2)	3,0 (1,0–6,5)	**< 0,001**
**Tipo de diálise** Hemodiálise Peritoneal Ambas Preemptivo	79 (79,8) 5 (5,0) 10 (10,1) 5 (5,0)	24 (80,0) 2 (6,6) 1 (3,3) 3 (10,0)	55 (79,7) 3 (4,3) 9 (13,0) 2 (2,9)	0,253
Doador vivo (%)	28 (28,2)	15 (50,0)	13 (18,8)	**0,003**
**Primeiro transplante renal (%)**	83 (83,8)	29 (96,6)	54 (78,2)	**0,035**
**Reatividade ao Painel** Mediana (P25–75) ≥ 80%	26,0 (2,8–60,0) 11 (11,1)	6,5 (0–17,0) 0	26,0 (3,5–65,5) 11 (15,9)	**0,039** 0,583
**Anticorpo anti–HLA doador** Sim 1 2	11 (11,1) 7 (7,1) 4 (4,0)	0 0 0	11 (15,9) 7 (10,1) 4 (5,8)	1,000
**Imunossupressão (%)** Timoglobulina Basiliximabe Ciclosporina Tacrolimus Azatioprina Micofenolato	40 (40,4) 30 (30,3) 25 (25,2) 73 (73,7) 20 (20,2) 79 (79,8)	0 4 (13,3) 23 (76,6) 6 (20,0) 18 (60,0) 12 (40,0)	40 (57,9) 26 (37,6) 2 (2,9) 67 (97,1) 2 (2,9) 67 (97,1)	**< 0,001** **0,018** **< 0,001** **< 0,001** **< 0,001** **< 0,001**
**Complicações no pós–transplante (%)** Infecções bacterianas Infecções virais Neoplasia Sangramento Cardiológica	49 (49,4) 27 (27,2) 12 (12,1) 7 (7,0) 6 (6,0)	10 (33,3) 3 (10,0) 6 (20,0) 0 5 (16,6)	39 (56,5) 24 (34,7) 6 (8,7) 7 (10,1) 1 (1,4)	0,359 0,519 **0,006** 0,589 **< 0,001**
Taxa de filtração glomerular* (mediana, P25–75) 1 ano 5 anos	60,0 (46,0–74,1) 49,5 (31,8–65,8)	64,5 (48,7–71,5) 50,0 (37,7–66,3)	59,7 (42,7–76,2) 45,6 (30,9–63,2)	0,576 0,331
**Razão proteinúria/creatinúria (mediana, P25–75)** 1 ano 5 anos	0,20 (0,10–0,40) 0,27 (0,15–0,66)	0,10 (0,10–0,30) 0,23 (0,10–0,50)	0,20 (0,15–0,50) 0,36 (0,15–0,79)	0,089 0,350
**Biópsia do enxerto renal** Sim Rejeição Recorrência de lúpus	46 (46,4) 23 (23,2) 5 (5,0)	15 (50,0) 5 (16,6) 4 (13,3)	31 (44,9) 18 (26,0) 1 (1,4)	0,504 0,301 **0,033**
**Acompanhamento pós–transplante renal (%)** Seguimento ambulatorial Perda do enxerto Óbito com enxerto funcionante Perda de seguimento	45 (45,5) 37 (37,4) 13 (13,1) 4 (4,0)	5 (16,6) 20 (66,7) 5 (16,6) 0	40 (58,0) 17 (24,6) 8 (11,6) 4 (5,8)	**< 0,001**
**Causa da perda do enxerto renal** Trombose vascular Rejeição Infecção	6 (6,0) 25 (25,2) 6 (6,0)	0 19 (63,3) 1 (3,3)	6 (8,7) 6 (8,7) 5 (7,2)	**0,009**
**Causa de óbito** Cardiovascular Infecção Neoplasia Outras	4 (4,0) 5 (5,0) 2 (2,0) 2 (2,0)	1 (3,3) 3 (10,0) 1 (3,3) 0	3 (4,3) 2 (2,9) 1 (1,4) 2 (2,9)	0,375

Abreviaturas – LES: lúpus eritematoso sistêmico; TR: transplante renal. Notas – *Equação CKD-EPI 2021 baseada em creatinina.

A mediana de tempo entre o diagnóstico de LES e o TR foi de 9,75 (2,83–16,12) anos, similar entre G1 e G2. A mediana de tempo entre o início da diálise e o TR foi de 1,5 (0,6–3,5) ano, similar entre os grupos. Pacientes do G1 receberam mais frequentemente um primeiro transplante (96,6% *vs*. 78,2%, p = 0,035) de doador vivo (50% *vs*. 18,8%, p = 0,003). A mediana da reatividade ao painel foi menor no G1 comparado ao G2 [6,5 (0–17,0) *vs*. 26,0 (3,5–65,5), p = 0,039].

### Manifestações Extrarrenais do LES

Em relação às manifestações extrarrenais do LES houve uma redução significativa de pacientes com queixas articulares [71,8 % *vs.* 52,5%, OR 0,70 (0,010–0,472), p < 0,001], mucocutâneas (fotossensibilidade e *rash* malar) [70,6 % *vs.* 52,5%, OR 0,026 (0,004–0.183), p < 0.001] e neurológicas (convulsões) [12,1 % *vs.* 8,1%, OR 0,046 (0,014–0,153), p < 0,001] antes e após o TR, respectivamente. Não houve diferença na frequência de eventos trombóticos, obstétricos, oftalmológicos e serosites (não mostrados). A presença de anticoagulante lúpico, anticorpo anticardiolipina ou de anticorpo anti-beta 2 glicoproteína 1 isolados ou em associação não esteve associada a trombose pré-transplante, trombose pós-transplante ou sangramento pós-transplante (não mostrados).

### Evolução Após o Transplante

As principais complicações pós-transplante foram infecções bacterianas (48,4%) e virais (26,2%), semelhantes entre os grupos. O desenvolvimento de neoplasias foi significativamente maior no G1 comparado ao G2 (20% *vs*. 8,7%, p = 0,006, respectivamente), destacando-se as relacionadas ao HPV (n = 5), mama (n = 3), linfoma (n = 2) e rim (n = 1). Doença cardiovascular também foi significativamente mais frequente no G1 comparado ao G2 (16,6% *vs*. 1,4%, p < 0,001).

A TFG em 1 ano (64,5 *vs*. 59,7 mL/min/1,73m^2^, p = 0,57) e em 5 anos (50,0 *vs*. 45,6 mL/min/1,73m^2^, p = 0,33), bem como a proteinúria em 1 ano (0,10 *vs*. 0,20, p = 0,08) e em 5 anos (0,23 *vs*. 0,36, p = 0,35) foi semelhante em ambos os grupos.

Houve indicação de biópsia do enxerto renal em 46% dos pacientes, com proporção semelhante de episódios de rejeição nos dois grupos (16,6% *vs*. 26,0%, p = 0,30). Houve recorrência de NL após o transplante em 4 (13,3%) pacientes do G1 e em 1 (1,4%) paciente do G2, p = 0,033. O tempo mediano para recorrência foi de 9,0 (5,5-10,0) anos, semelhante entre os grupos (p = 0,051). Os 4 pacientes do G1 com recorrência perderam função do enxerto e retornaram à diálise, em uma mediana de 2,5 (0,5–6,7) anos, com biópsias subsequentes demonstrando rejeição. A paciente do G2 com recorrência de NL encontra-se em acompanhamento (5 anos após recorrência), com TFG de 45 mL/min/1,73m^2^ e RPC 0,1. Não foram identificados outros fatores significativamente associados com recorrência, exceto o período do transplante: idade, sexo, cor de pele, presença de autoanticorpos pré-transplante, tipagem HLD-DR, cPRA, imunossupressão, tipo de doador, rejeição.

### Sobrevida do Paciente e do Enxerto Renal

A mediana de tempo de acompanhamento após TR foi 5,0 (1,7–10,4) anos, maior no G1 comparado ao G2 (14,0 *vs.* 3,0, p < 0,001), como esperado por ser a coorte mais antiga. Perda do enxerto ocorreu em 37 (37,4%) pacientes e óbito com enxerto funcionante em 13 (13,1%) durante todo o acompanhamento. As causas de perda do enxerto renal foram trombose vascular (n = 6), infecção (n = 6), rejeição (n = 25). Nenhuma perda de enxerto foi atribuída à recorrência de NL. Dentre as causas de óbito com enxerto funcionante, 4 foram cardiovasculares, 2 por neoplasias, 5 por infecção, sendo 4 destas em decorrência de infecção por SarsCov2.

A sobrevida do enxerto em 5 anos foi 73,6%, semelhante à dos pacientes transplantados por outras causas (71,5%, p = 0,308) (não ilustrado). As curvas de sobrevida do paciente e do enxerto estão representadas nas Figuras 1 e 2, respectivamente. Não houve óbitos em 5 anos no G1, e a sobrevida do paciente no G2 foi de 87%. A sobrevida do enxerto em 5 anos foi similar entre os grupos (86,7% *vs.* 67,5%, p = 0,054). As sobrevidas do paciente e do enxerto foram significativamente maiores no G1 comparado ao G2 em 10 anos (96,2% *vs.* 65,3%, p = 0,018 e 66,7% *vs.* 37,9%, p = 0,025, respectivamente), e no tempo total de acompanhamento (51,1% *vs.* 65,3%, p = 0,025 e 53,3% *vs.* 37,9%, p = 0,043, respectivamente). Na análise pela regressão de Cox, não foram identificados fatores associados a perda de enxerto renal ou a óbito em 5 anos, 10 anos ou em todo o acompanhamento (testados sexo, idade no transplante, tipo de diálise, tipo de doador, cPRA, presença de anticorpo anti-HLA, tipagem HLA-DR, episódios de rejeição, recorrência de NL, TFG em 1 ano e RPC em 1 ano).

**Figura 1:** Sobrevida do paciente após transplante renal por nefrite lúpica.

**Figura 2:** Sobrevida do enxerto após transplante renal por nefrite lúpica.

## Discussão

Este estudo foi conduzido em um centro único, avaliando evolução, complicações e sobrevida de 99 pacientes que receberam transplante renal devido à nefrite lúpica (NL) entre 1977 e 2023. Foram comparados dois grupos, divididos pela mudança de imunossupressão, antes de 2009 (G1) e a partir de 2009 (G2), com função do enxerto e sobrevida comparáveis em 5 anos. A indicação de biópsia do enxerto renal foi semelhante nos grupos, com maior recorrência de NL no G1. A recorrência da NL documentada por biópsia não esteve associada à perda do enxerto renal.

Estudos de prevalência colocam o diagnóstico de NL em mulheres entre a terceira e quarta décadas de vida, com maior frequência entre afro-americanos e afro-caribenhos e maior severidade da doença renal em negros e hispânicos^1,3^. A população do nosso estudo consistiu-se majoritariamente de mulheres brancas com diagnóstico de LES entre a segunda e terceira décadas de vida, submetidas a TR entre a terceira e quarta décadas de vida. Essa diferença étnica reflete características específicas da nossa população local^9^.

A principal comorbidade foi a hipertensão arterial sistêmica, semelhante ao encontrado em outros estudos^8,10^. A hipertensão está associada à gravidade da lesão renal e ao tipo de lesão histológica, mas também à desregulação imune e à ativação do sistema renina-angiotensina-aldosterona, ao uso de corticoides e à aterosclerose acelerada pelo estado inflamatório crônico^10^.

Diversos fatores clínicos e demográficos foram investigados e associados à evolução da NL antes e após o transplante renal. Sexo masculino foi descrito como de maior risco para progressão da NL^3,11^, e no presente estudo não esteve associado a tempo entre diagnóstico de LES e TR nem influenciou sobrevida. As tipagens HLA-DR3 e HLA-DR15 foram associadas à apresentação desfavorável da NL^3,5^, porém sem associação com sobrevida do enxerto^12^. Os sorotipos HLA-DR4 e DR11 são descritos como fatores de proteção para o desenvolvimento de NL^3,5,13^. No nosso estudo, encontramos uma frequência significativa desses alelos, mas não identificamos uma associação direta com o desfecho clínico.

A forma de terapia renal substitutiva (hemodiálise *versus* diálise peritoneal) não impactou na sobrevida, em consonância com uma revisão publicada em 2020, comparável à de pacientes em diálise por outras causas^3^. O TR traz melhor qualidade de vida e maior sobrevida em comparação à diálise em diversos cenários^14,15,16^, inclusive na NL^3^, com redução da mortalidade por todas as causas e por doença cardiovascular, doença coronariana, infecção e sepse em relação aos pacientes que não foram transplantados^8^.

As principais manifestações extrarrenais do LES tendem a desaparecer nos pacientes em diálise, inclusive com negativação dos marcadores de atividade imunológica, permitindo inclusive a retirada dos imunossupressores^17^. Um recente estudo de Kim et al., no entanto, observou que até um quarto dos pacientes com NL em diálise apresentaram exacerbações do LES, principalmente manifestações hematológicas e constitucionais^18^. Aproximadamente metade dos pacientes do nosso estudo apresentava marcadores imunológicos negativos durante a avaliação para TR, e as principais manifestações extrarrenais do LES foram mucocutâneas, articulares e hematológicas, que reduziram significativamente em frequência após o TR.

O risco de desenvolvimento de falência renal na NL depende de diversos fatores, inclusive classe histológica, o acesso à saúde e a resposta aos tratamentos imunossupressores^3,19^, sendo o controle da proteinúria o principal parâmetro de controle de doença e definidor de resposta parcial ou completa, apesar de não haver consenso nos valores-alvo^3^. Uma revisão sistemática e meta-análise publicada em 2016 identificou redução do risco de progressão para falência renal ao longo das décadas, possivelmente relacionada ao desenvolvimento de protocolos de imunossupressão, mas que estagnou nos últimos anos. O risco em países desenvolvidos é de 10%, 15% e 20% em 5, 10 e 15 anos, respectivamente, sendo até 10% maior em países em desenvolvimento^6^. A evolução no tratamento da NL foi demonstrada neste estudo, com um predomínio do uso de azatrioprina nas primeiras décadas, passando para micofenolato no período mais recente, com a introdução de inibidores de calcineurina e rituximabe.

A NL classe IV está associada a maior risco de progressão para falência renal, chegando a 44% em 15 anos, cifra duas vezes superior ao risco na NL classe V^6^. Portanto, a identificação da classe histológica tem importância na estratificação de risco e no tratamento da NL. Em contrapartida, para pacientes com evolução para estágio final de doença renal não há benefício na realização de biópsia renal, pois os achados histopatológicos serão compatíveis com rim terminal, sem mudanças de conduta no que concerne ao tratamento renal substitutivo^20^. No presente estudo, a documentação da classe histológica foi limitada, com a maioria dos pacientes apresentando classe IV, conforme esperado para pacientes com falência renal. Podemos atribuir essa ausência de registro ao fato de os pacientes terem sido referenciados para o TR, já em tratamento dialítico, após o acompanhamento e tratamento em outros centros, sendo possivelmente negligenciada a importância dessa informação no contexto de falência renal.

As medianas de tempo entre o diagnóstico de LES e o transplante renal e entre o início da diálise e o transplante foram semelhantes entre os grupos G1 e G2. No entanto, o G1 recebeu mais frequentemente transplantes de doador vivo (50% *vs.* 18,8%), em consonância com o demonstrado no Registro Brasileiro de Transplantes ao longo do tempo^21^. O tipo de doador tem impacto na sobrevida do paciente e do enxerto^21^ e nas complicações pós-transplante, mas não foi identificada tal associação no presente estudo.

Um dos fatores relacionados à perda precoce do enxerto é a presença de anticorpos antifosfolípides^3^, frequente em pacientes com LES, apesar de o desenvolvimento da síndrome antifosfolípide (SAF) ser menos comum. Ames et al. demonstraram que pacientes com anticorpos antifosfolípides apresentam maior risco de eventos trombóticos, incluindo microangiopatia trombótica no enxerto, devendo idealmente ser rastreados antes da realização do transplante^22^. A política de rastreio de SAF é dispendiosa, e no nosso centro é realizada em pacientes com fatores de risco identificáveis adicionais ao diagnóstico de LES, como eventos de trombose prévia ou de abortamentos. A frequência de positividade de anticorpos antifosfolípides no nosso estudo foi baixa; não foi encontrada associação estatisticamente significativa entre a presença desses anticorpos e trombose ou perda do enxerto no pós-transplante. Possivelmente não houve poder estatístico para demonstrar essa associação, mas também devemos ressaltar que, em pacientes com identificação de maior risco de trombose, protocolarmente é iniciada anticoagulação perioperatória.

O estado pró-inflamatório relacionado ao LES, doenças valvares, presença de anticorpos antifosfolípides e tratamentos imunossupressores prévios podem elevar o risco de complicações infecciosas, cardíacas e cerebrovasculares após o transplante renal^4,23^. A corticoterapia prolongada no pré e pós-TR pode agravar doenças cardiovasculares e predispor a osteoporose, diabetes *mellitus*, catarata, miopatias, entre outras. As infecções também parecem ser mais frequentes e ter maior severidade em pacientes com LES, em especial nos que receberam imunossupressão prolongada pré-TR^24^. Neste estudo, as principais complicações apresentadas foram infecções bacterianas e virais, seguidas por neoplasias e, menos frequentes, cardiológicas.

O risco de desenvolvimento de neoplasias malignas está associado ao uso de imunossupressores antes do transplante em geral^25,26^, e os pacientes com LES apresentam risco aumentado de neoplasias em comparação com a população geral independentemente do TR^4,27,28^. No entanto, estudos demonstraram que pacientes com NL que receberam transplante renal parecem ter os mesmos riscos de desenvolver malignidades (exceto melanoma) que os pacientes que receberam transplante renal por outras causas^29^. No presente estudo, a frequência de neoplasias foi superior à encontrada em outro levantamento da instituição^30^, a maioria relacionada ao HPV.

A função renal em 1 ano e em 5 anos foi semelhante nos dois grupos, com imunossupressões distintas.

A recorrência de NL pós-TR é estimada em 2% a 11%^3,31,32,33^; podendo chegar a mais de 50% quando em biópsias protocolares^34^. A apresentação mais frequente é classe II, e sem impacto na sobrevida do enxerto^3,32,34^. A recorrência de NL em nossa amostra foi de 5%, e a demonstração de classes IV, V e VI se justifica pelo fato de que as biópsias foram indicadas por disfunção do enxerto ou proteinúria, e não protocolares. A mediana de tempo para recorrência foi de 9 anos, e nenhuma perda do enxerto renal foi atribuída à recorrência de NL. Houve maior frequência de recorrência no grupo com maior tempo de acompanhamento, mas não foi identificada associação com a imunossupressão.

A perda do enxerto ocorreu em 37,4% dos pacientes, e as causas principais foram rejeição e infecção. Houve óbito com enxerto funcionante de 13% dos pacientes, principalmente por causa cardiovascular, infecção e neoplasias.

Existem divergências quanto à sobrevida do enxerto de pacientes com NL em relação aos TR por outras causas, com estudos demonstrando resultados inferiores^35,36,37,38,39^ e outros com sobrevidas comparáveis^40,41,42,43,44^ e mesmo superiores^12^. Nosso estudo demonstrou sobrevida do enxerto renal em 5 anos comparável aos transplantados por outras causas e aos registros históricos dos TR da instituição^45,46^ em diferentes cenários. Entretanto, em 10 anos e ao longo de todo o seguimento, a sobrevida dos pacientes e do enxerto renal foi superior no G1 comparado ao G2. Não foram identificados fatores significativamente associados a essa diferença, sendo avaliados sexo, idade no transplante, tipo de diálise, tipo de doador, cPRA, presença de anticorpo anti-HLA, tipagem HLA-DR, episódios de rejeição, recorrência de NL, TFG em 1 ano e proteinúria em 1 ano. Nota-se que o número de pacientes do G2 que atingiram tempo de seguimento de 10 anos ou mais foi muito pequeno, o que pode explicar a diferença de sobrevida.

As principais limitações do estudo estão relacionadas ao seu caráter retrospectivo, compreendendo um longo período de tempo, que pode ter comprometido a acurácia de algumas informações clínicas especialmente antes da implementação do prontuário eletrônico. Entre os pontos positivos do estudo, destaca-se o grande número de pacientes transplantados renais por NL na Instituição e o longo período de seguimento fornecem dados valiosos sobre desfechos em longo prazo.

Concluindo, a NL é uma manifestação grave do LES, levando à falência renal. O transplante renal é um tratamento bem-sucedido para esses pacientes, com taxas de sobrevida do enxerto semelhantes às de pacientes transplantados por outras causas. A recorrência de NL não esteve associada à perda do enxerto renal.

## Contribuição dos Autores

BCP, LB, GM e EK conduziram a conceitualização, curadoria, análise, administração de dados, redação e revisão do manuscrito. LB e HB contribuíram para a metodologia, administração e curadoria de dados. VDG contribuiu com a revisão e aprovação final do manuscrito. Todos os autores contribuíram substancialmente para o desenvolvimento deste manuscrito.
